# The HDAC Inhibitor Vorinostat Diminishes the *In Vitro* Metastatic Behavior of Osteosarcoma Cells

**DOI:** 10.1155/2015/290368

**Published:** 2015-02-17

**Authors:** Xiaodong Mu, Daniel Brynien, Kurt R. Weiss

**Affiliations:** Laboratory of Cancer Stem Cell, Stem Cell Research Center, University of Pittsburgh, Pittsburgh, PA 15217, USA

## Abstract

Osteosarcoma (OS) is the most common primary malignancy of bone and affects patients in the first two decades of life. The greatest determinant of survival is the presence of pulmonary metastatic disease. The role of epigenetic regulation in OS, specifically the biology of metastases, is unknown. Our previous study with the murine OS cell populations K7M2 and K12 demonstrated a significant correlation of metastatic potential with the DNA methylation level of tumor suppressor genes. In the current study, we investigated if the histone deacetylase (HDAC) inhibitor, vorinostat, could regulate the metastatic potential of highly metastatic OS cells. Our results revealed that vorinostat treatment of highly metastatic K7M2 OS cells was able to greatly reduce the proliferation and metastatic potential of the cells. Morphological features related to cell motility and invasion were changed by vorinostat treatment. In addition, the gene expressions of mTOR, ALDH1, and PGC-1 were downregulated by vorinostat treatment. These data suggest that vorinostat may be an effective modulator of OS cell metastatic potential and should be studied in preclinical models of metastatic OS.

## 1. Introduction

Osteosarcoma (OS) is the most common primary malignancy of bone and usually presents during the first two decades of life. Current treatment protocols include neoadjuvant chemotherapy, surgical resection, and postoperative chemotherapy. Five-year overall survival in patients without metastatic disease is 65–70%. In patients with pulmonary metastases at the time of diagnosis, however, the survival rate is only 15–30%. These statistics have not changed appreciably in nearly thirty years, and pulmonary metastases remain the major determinant of OS mortality [1–7]. Therapies designed to target metastatic disease provide the potential for novel OS treatment strategies but are not widely available at the present time. The greatest obstacle to the improvement of OS prognosis is the inability to effectively target and prevent pulmonary metastases [5, 8–10]. Better understanding of the biochemical mechanisms that drive OS metastatic potential is clearly necessary.

K7M2 and K12 are related cell populations derived from a spontaneously-occurring murine OS. K7M2 metastasizes violently to the lung in the mouse model of OS, whereas K12 is much less metastatic [9, 10]. We have published that K7M2 and K12 produce different quantities of cytokines and that inhibition of these cytokines alters OS cell behavior* in vitro* [11]. More recently we have demonstrated important differences between K7M2 and K12 in terms of the cancer stem cell factors mammalian target of rapamycin (mTOR), Notch1, and aldehyde dehydrogenase (ALDH1) [12–14]. As K7M2 and K12 are related but vary in their metastatic rates, they are powerful tools through which the qualities that confer metastatic potential may be elucidated.

Epigenetics (Greek: epi-over, above, outer) is the study of changes in gene expression or cellular phenotype caused by mechanisms other than changes in the underlying DNA sequence. Epigenetics has thus been called “the code outside the code.” Examples of epigenetic modification include DNA methylation and histone modification, both of which regulate gene expression but do not alter the genetic code.

Cancer has genetic and epigenetic origins. The epigenetic silencing of tumor suppressor genes is associated with tumor formation and progression. Epigenetic reprogramming of somatic cells to attain stem-like properties has been experimentally achieved by exposure of cells to an embryonic microenvironment. Exposure to an embryonic microenvironment can also exert a profound effect by epigenetically reprogramming tumor cells. We demonstrated these phenomena by treating K7M2 OS cells with chick embryo extract (CEE). We observed the dose-dependent reversal of methylation in the tumor suppressor genes p53, p16, and E-cadherin. We also appreciated alterations in K7M2 cell morphology, invasiveness, and resistance to oxidative stress that indicated decreased metastatic potential in CEE-treated cells [15].

Histone deacetylases (HDACs) are a family of enzymes involved in epigenetic modification. Binding of an acetyl group to a histone tail relaxes the chromatin in that region of DNA, allowing for increased gene expression. HDACs remove these acetyl groups, which tightens the chromatin around the histone and decreases gene expression. HDACs also interact with other epigenetic modifiers such as DNA binding proteins and methyl-binding proteins to further modify gene expression. HDACs have been shown to interact with transcription factors such as p53 and NF-kB. HDAC activity has been implicated in tumorigenesis, and HDAC has thus become a subject of ongoing oncological investigation [16].

As expected from its complex role in epigenetics, the inhibition of HDAC has been shown to alter the expression of a number of genes, some of which have been correlated with the tumorigenesis. Suberoylanilide hydroxamic acid (SAHA; vorinostat) is an inhibitor of Classes I and II HDAC that binds directly to the zinc atom in the enzyme's catalytic site [16]. Vorinostat has already been approved by the US Food and Drug Administration for use in patients with cutaneous T-cell lymphoma, and its potential use in other neoplasia is being actively investigated. Encouraged by our findings with CEE treatment, we wished to investigate the effects that vorinostat treatment would exert on K7M2 OS cells* in vitro*.

## 2. Materials and Methods

### 2.1. Cell Proliferation Assay

5000 K7M2 murine OS cells were cultured in 12-well plates with proliferation medium (PM; DMEM with 10% fetal bovine serum (FBS) and 5% penicillin and streptomycin, Invitrogen Life Technologies, Grand Island, NY). Inhibition of HDAC was achieved through vorinostat at concentrations of 0 *μ*M, 25 *μ*M, 50 *μ*M, and 100 *μ*M. The cells were incubated in the presence of vorinostat for 48 hours.

### 2.2. Actin Staining

Organization of the actin cytoskeleton in K7M2 OS cells was assessed using the phalloidin stain conjugated with Alexa Fluor 488 (Invitrogen Life Technologies, Grand Island, NY). Cells were washed twice with phosphate-buffered saline (PBS), fixed in 3.7% formaldehyde for 10 minutes at room temperature, and washed twice more with PBS. Cells were then permeabilized in 0.1% Triton X-100 for 20 minutes and washed again with PBS. For each well, a staining solution of 5 *μ*L of ethanol stock solution phalloidin with 200 *μ*L PBS and 1% bovine serum albumin was added. The staining solution was kept in wells for 20 minutes and then washed again with PBS.

### 2.3. *In Vitro* Cell Invasion Assay

*In vitro* cell invasion was assessed using a real-time cell invasion and migration (RT-CIM) assay system (ACEA Biosciences, Inc, San Diego, CA) with a 16-well transwell plate (CIM-plate 16, Roche Diagnostics, Mannheim, Germany). The surfaces of the upper wells were coated in 5% Matrigel (BD BioSciences, Bedford, MA), and 10% FBS-containing PM was added to the lower chambers. Cells in serum-free medium were added to the upper chambers, and the migration of the cells was monitored by the system every 15 minutes for 24 hours. Data analysis was performed by the supplied RTCA 1.2 software (Roche Diagnostics, Mannheim, Germany) supplied with the instrument.

### 2.4. mRNA Analysis with Reverse Transcriptase Polymerase Chain Reaction (RT-PCR)

Total RNA was obtained from K7M2 cells using the RNeasy Mini Kit (Qiagen, Inc., Valencia, CA) according to the manufacturer's instructions. Reverse transcription was performed using the iScript cDNA Synthesis Kit (Bio-Rad Laboratories, Inc., Hercules, CA). The sequences of primers are given in Table 1 and include Notch1, mTOR, ALDH1, microtubule-associated protein 1A/1B-light chain 3 (LC3), peroxisome proliferator-activated receptor gamma coactivator 1 (PGC-1), and the housekeeping gene glyceraldehydes 3-phosphate dehydrogenase (GAPDH). PCR reactions were performed using an iCycler Thermal Cycler (Bio-Rad Laboratories, Inc., Hercules, CA). The cycling parameters used for all primers were as follows: 95°C for 10 minutes; PCR, 40 cycles of 30 seconds at 95°C for denaturation, 1 minute at 54°C for annealing, and 30 seconds at 72°C for extension. Products were separated by size and were visualized on 1.5% agarose gel stained with ethidium bromide. All data were normalized to the expression of GAPDH.

## 3. Results

### 3.1. Cell Proliferation Assay

**Figure 1:**
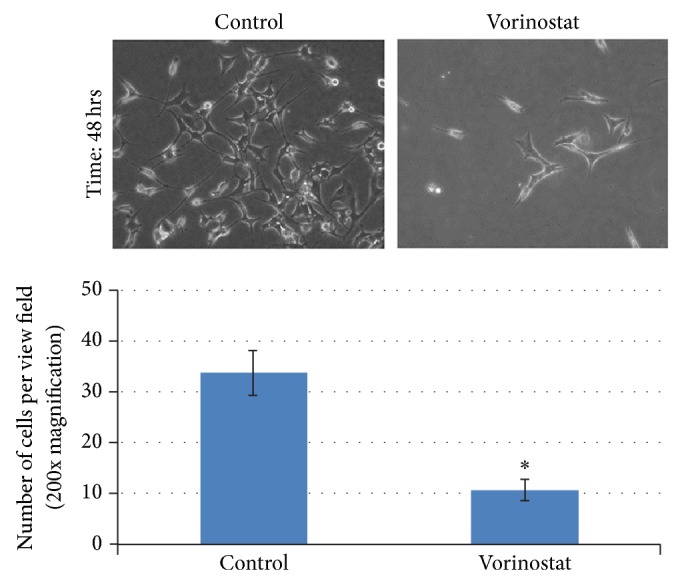
A significant decrease in K7M2 cell proliferation with vorinostat treatment (100 *μ*M for 48 hours).

Qualitative analysis of cell proliferation showed significant differences between the untreated and vorinostat-treated (100 *μ*M) cells. Vorinostat-treated cells displayed diminished proliferation and increased cell death, resulting in reduced cell number (Figure 1). Additionally, vorinostat-treated cells featured a more polygonal shape and less apparent invadopodia than control cells (Figure 1). These results were quantitatively similar to the cytoskeletal changes we previously appreciated with CEE and disulfiram treatment* in vitro*.

### 3.2. Actin Staining

**Figure 2:**
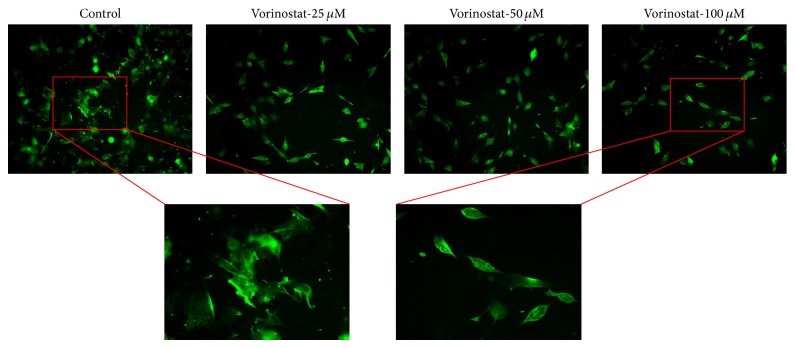
Morphological changes in the K7M2 actin cytoskeleton with increasing doses of vorinostat. Note expanded images of the control and vorinostat 100 *μ*M cells. Vorinostat treatment caused the cells to become polygonal, become regular, and lose their invadopodia.

Qualitative analysis showed morphological differences with increasing concentrations of vorinostat (Figure 2). Untreated K7M2 cells displayed characteristic elaborate actin cytoskeletal features of K7M2 cells that have been previously described: they are large and irregular and feature numerous invadopodia (Figure 2). The cells become increasingly polygonal with fewer invadopodia as the concentration of vorinostat increases.

### 3.3. *In Vitro* Cell Invasion Assay

**Figure 3:**
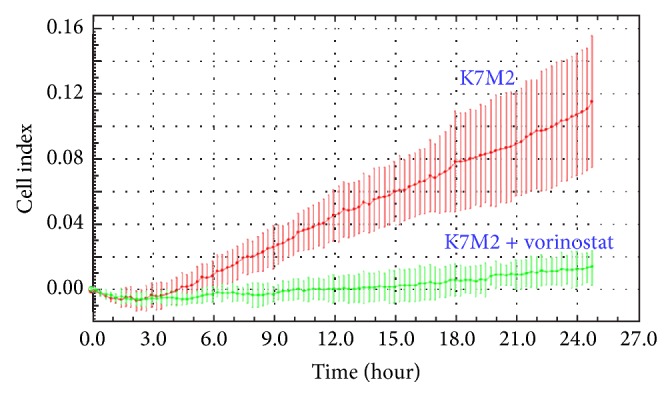
Invasion assays of untreated and vorinostat-treated (50 *μ*M) K7M2 cells through a semisolid matrigel matrix. There was a significant inhibition of invasion in vorinostat-treated cells.

Migration of vorinostat-treated (50 *μ*M) K7M2 cells through a semisolid Matrigel matrix was significantly reduced compared with untreated cells, as measured by a significant reduction in cell index (Figure 3).

### 3.4. RT-PCR

**Figure 4:**
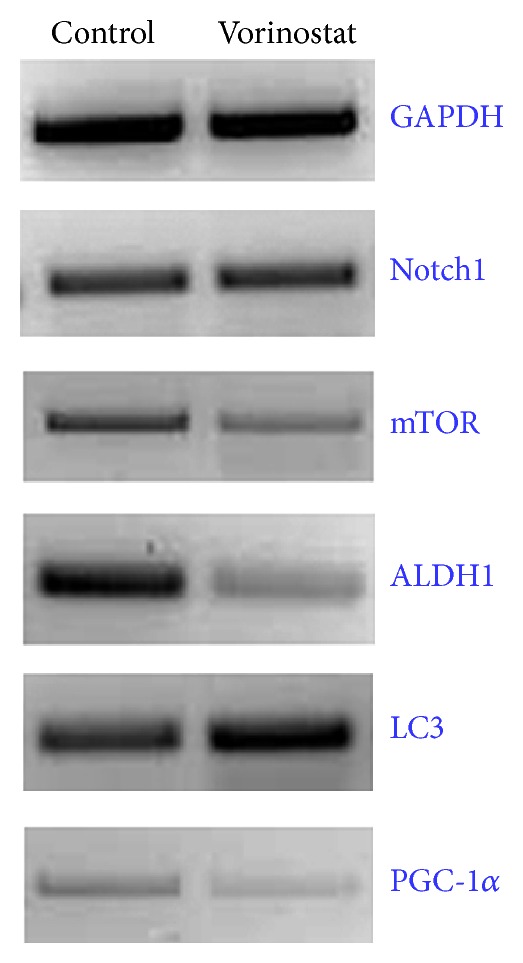
Differences in gene expression of K7M2 cells with vorinostat (50 *μ*M) treatment. The expressions of mTOR, ALDH, and PGC-1 were all decreased, whereas Notch1 expression did not change and LC3 expression increased with vorinostat treatment.

RT-PCR was used to analyze the expressions of several genes of interest. We have previously described the enhanced expressions of mTOR, ALDH1, and Notch1 in K7M2 OS cells compared with less metastatic K12 cells, indicating that they are metastasis-associated factors in OS. We have also demonstrated that inhibition with rapamycin, disulfiram, and DAPT, respectively, diminished their* in vitro* metastatic phenotypes. Vorinostat (50 *μ*M) treatment for 48 hours decreased mTOR and ALDH gene expressions but did not appear to affect Notch1 expression. LC3, a marker of autophagy, showed increased expression after vorinostat treatment. PGC1, a regulator of energy metabolism and mitochondrial biosynthesis, showed decreased expression with vorinostat treatment (Figure 4).

## 4. Discussion

The presence of pulmonary metastases in OS is the ultimate determinant of mortality for these patients, making metastatic disease an essential therapeutic target. To this end, agents and strategies that diminish the metastatic phenotypes of OS cells would represent a tremendous advancement in the treatment of OS. This study, despite its limitations, illustrates that vorinostat alters K7M2 cells genetically, morphologically, and behaviorally in a manner consistent with our previous observations of reduced metastatic potential.

Qualitative analysis of cell proliferation illustrated a decrease in cell proliferation with increasing concentrations of vorinostat. The fact that vorinostat affected K7M2 cells in a manner similar to CEE treatment is intriguing, as these epigenetic agents function by completely different mechanisms: demethylation versus histone deacetylase inhibition.

Actin staining was performed to visualize morphological changes of K7M2 cells after vorinostat treatment. We have previously observed that morphological changes in treated K7M2 cells correspond with differences in their metastatic phenotypes. Commensurate with our observations after CEE, disulfiram (ALDH1 inhibitor), rapamycin (mTOR inhibitor), and DAPT (Notch inhibitor) treatment, vorinostat treatment caused a dose-dependent alteration in K7M2 cell morphology [12–15]. These changes included the adoption of a more regular polygonal shape and the diminution of invadopodia, which are associated with greater motility and thus metastatic potential.

These observations were corroborated with a matrigel invasion assay. Vorinostat treatment caused a powerful inhibition of OS cells' ability to migrate through a three-dimensional matrigel matrix. In our opinion, this result provides the strongest evidence that vorinostat treatment diminishes the metastatic potential of K7M2 cells* in vitro*. In order to metastasize, OS cells must necessarily migrate out of the tumor and enter the bloodstream and then migrate back out of the bloodstream to initiate growth in the lungs. The observation that vorinostat inhibited this capacity so potently is encouraging.

PCR was utilized to evaluate the expression of genes previously found to be associated with OS metastatic potential, autophagy, and metabolism. Somewhat surprisingly, Notch expression was unaltered by vorinostat treatment in this study. In previous work, we found that Notch1 inhibition did not cause changes in cell proliferation but did result in diminished invasion capacity and resistance to oxidative stress [13]. Perhaps the decrease in proliferation we observed was independent of Notch1 activity.

mTOR also has a complex role in OS development that we hypothesize, based on our previous work, is linked to cell migration. Suppression of the mTOR pathway by any means could, therefore, lead to decreases in cell migration. The PCR results indeed suggested decreased mTOR expression with vorinostat treatment, a finding which might help to explain the changes in K7M2 cell morphology and invasiveness that we observed.

ALDH1 is a cancer stem cell-related factor that allows cells of all types to withstand the effects of oxidative stress [8, 17–23]. That ALDH expression was decreased with vorinostat treatment is intriguing. The next logical step is to investigate if vorinostat treatment causes K7M2 cells to display reduced resistance to oxidative stress. These experiments are underway. We have demonstrated in a small clinical series of bone sarcoma patients that the clinical event of metastasis correlates positively with ALDH1 activity. Our data suggest that vorinostat may alter cell morphology and invasiveness through an ALDH1-associated pathway. We are currently evaluating the feasibility of disulfiram as an adjuvant to OS treatment. Perhaps this would be an even more potent approach if vorinostat and disulfiram were used in combination. These results should be investigated more completely in future studies.

We also evaluated the expressions of LC3 and PGC1. LC3 is an intracellular protein that is critically important to the process of autophagy [24]. The dramatic increase in LC3 may provide a clue as to the mechanism of cell death we appreciated with microscopy. PGC1 is involved with mitochondrial biosynthesis [25]. Our observation of dramatically decreased PCG1 expression indicates that vorinostat treatment may cause a decrease in cellular respiration and metabolism. This would certainly account for the morphological and behavioral phenomena we appreciated with vorinostat treatment. This is another area of investigation that ought to be pursued.

There are several limitations to this study. Chief among these limitations are the facts that these experiments were performed entirely* in vitro* with a single murine OS cell population. Furthermore, we do not yet understand which HDAC subtypes are most active in OS.

That being the case, the results described here clearly lend support to the small but growing body of literature regarding OS and the logic of epigenetic modulation. In a disease that has not witnessed a significant prognostic improvement for nearly thirty years, epigenetic modulation with vorinostat may represent a novel way to target OS metastatic biology. Future studies will focus on a more complete understanding of vorinostat's mechanisms and pathways of action. Specifically, it is not known which HDAC subtype(s) are most important in sarcoma generally and OS specifically. Studies such as these may help to maximize the efficacy of HDAC inhibition in sarcoma. Certainly, these data support the further investigation of HDAC inhibition in OS in our preclinical murine model of OS, or other preclinical models of OS.

## Figures and Tables

**Table 1:** PCR primer sequences.

Gene	Primer sequence
GAPDH	Forward: TCCATGACAACTTTGGCATTG
	Reverse: TCACGCCACAGCTTTCCA

Notch1	Forward: GCCGCAAGAGGCTTGAGAT
	Reverse: GGAGTCCTGGCATCGTTGG

mTOR	Forward: CAGTTCGCCAGTGGACTGAAG
	Reverse: GCTGGTCATAGAAGCGAGTAGAC

ALDH1	Forward: GACAGGCTTTCCAGATTGGCTC
	Reverse: AAGACTTTCCCACCATTGAGTGC

LC3	Forward: CGCTTGCAGCTCAATGCTAAC
	Reverse: TGCCCATTCACCAGGAGGA

PGC-1*α*	Forward: CGGAAATCATATCCAACCAG
	Reverse: TGAGGACCGCTAGCAAGTTTG
